# Development of a Remote Psychological First Aid Protocol for Healthcare Workers Following the COVID-19 Pandemic in a University Teaching Hospital, Malaysia

**DOI:** 10.3390/healthcare8030228

**Published:** 2020-07-24

**Authors:** Ahmad Hatim Sulaiman, Zuraida Ahmad Sabki, Mohd Johari Jaafa, Benedict Francis, Khairul Arif Razali, Aliaa Juares Rizal, Nor Hazwani Mokhtar, Johan Arif Juhari, Suhaila Zainal, Chong Guan Ng

**Affiliations:** 1 Department of Psychological Medicine, Faculty of Medicine, University of Malaya, Kuala Lumpur 50603, Malaysia; zuraidasabki@um.edu.my (Z.A.S.); aliaajuares@gmail.com (A.J.R.); johan.ariff@ummc.edu.my (J.A.J.); chong_guan@um.edu.my (C.G.N.); 2 Psychology and Counselling Management Unit, University Malaya Medical Centre, Kuala Lumpur 59100, Malaysia; johari@ummc.edu.my (M.J.J.); suhailaza@ummc.edu.my (S.Z.); 3 Department of Psychological Medicine, University Malaya Medical Centre, Kuala Lumpur 59100, Malaysia; benedict@ummc.edu.my (B.F.); khairularif.r@ummc.edu.my (K.A.R.); wani@ummc.edu.my (N.H.M.)

**Keywords:** COVID-19, psychological crisis intervention, remote Psychological First Aid, healthcare workers, SMART

## Abstract

The purpose of this article is to discuss the importance of addressing the psychological impact of coronavirus disease 2019 (COVID-19) on healthcare workers (HCWs) who are frontliners directly involved in mitigating the spread of the disease. This paper focuses on the utilization of a clinical practice protocol for identifying HCWs who are COVID-19-positive or under investigation and surveillance for suspected infection, in a tertiary, university teaching hospital of Malaysia. The protocol for Psychological First Aid (PFA), which is applied remotely via a mobile application and phone calls, outlines the work process in stages, with expected immediate, intermediate, and long-term goals within a “Specific, Measurable, Attainable, Relevant, and Realistic Timeframe” (SMART). This protocol is developed to provide a guideline for psychological crisis interventions that promote safety, calm, and hope in HCWs, allowing them to return to psychological functioning without being stigmatized. The unprecedented remote PFA protocol may serve as a platform for further research on the application of a goal-directed approach in a healthcare organization.

## 1. Introduction

*“Pandemic is not a word to use lightly or carelessly. It is a word that, if misused, can cause unreasonable fear, or unjustified acceptance that the fight is over, leading to unnecessary suffering and death.”*

[1]

On 11 March 2020, the World Health Organization (WHO) Director General Dr Tedros Adhanom Ghebreyesus declared the novel coronavirus disease 2019 (COVID-19) outbreak to be a global pandemic, following an exponential increase in the number of cases and fatalities outside China [1] and its rapid spread to South East Asia countries including Malaysia. On 18 March 2020, the Malaysian government imposed the Movement Control Order under the Prevention and Control of Infectious Diseases Act 1988 and Police Act 1967 [2]. The stringent lockdown measures, including the practice of social distancing and hand hygiene, has helped to mitigate the spread of COVID-19, flattening the pandemic curve, hence reducing the number of patients requiring intervention at the same time and averting the health care system from exceeding its capacity [3]. It is imperative that the health authorities overestimate the potential biological and psychological impact of this novel and highly contagious outbreak through early identification and isolation of suspected cases. This has resulted in more healthcare workers (HCWs) being deployed to combat the pandemic while potentially exposing them to deleterious effects on their physical and mental health. These workers are subjected to long working hours using highly protective personal equipment that can contribute to stress, fatigue, occupational burn-out, fear, panic, worry, and emotional distress [4,5]. Having to serve while self-protecting against infection, risking, eventually, to “lose workplace control and autonomy” also contributes to their psychological crisis [6]. 

There is a limited number of publications and accurate national situation reports on the number of infections and deaths among healthcare workers. In China, 3.8% of infections were among healthcare workers, with five deaths [7]. The International Council of Nurses reported that more than 260 nurses succumbed to COVID-19, while at least 90,000 HCWs were infected worldwide [8]. Preventing the transmission of the disease through risk assessment tools and protocols for healthcare providers is as equally important as having psycho-social support services to provide psychological crisis interventions and evaluation for stress, anxiety, depression, and burnout among them [9].

### 1.1. University Malaya Medical Centre as the COVID-19 Healthcare Provider

The government has identified 26 government hospitals under the Ministry of Health (MoH) and University Malaya Medical Centre (UMMC, under the purview of the Ministry of Higher Education) as designated COVID-19 hospitals. UMMC is the first non-MoH teaching hospital to be listed due to its reputable healthcare services and its location at the micro epicenter of the epidemic in the Klang Valley. 

UMMC COVID-19 Task Force was formed following the gazettement led by the dean of the Faculty of Medicine, University of Malaya (UM), and consists of clinical and non-clinical experts, researchers, administrative personnel, and nurses. It has synergistically and strategically developed and improvised hospital policies with regard to ambulatory health services and day-care services for oncology and elective surgeries. The hospital vigorously has responded through planned relocation of in-patients and upgraded the ward layout, especially, the Intensive Care Units and COVID wards. The HCWs directly affected by this restructuring are physicians (infectious disease and chest specialists, emergency medicine doctors, anesthesiologists), medical officers deployed to run COVID-19 swab tests, nurses, disinfectant teams, cleaners, and others. These are the frontliners who are at risk of infection while working and risk being overwhelmed by physical and psychological constraints due to work demands.

The Task Force has unanimously acknowledged the psychological impact of the COVID-19 outbreak on HCWs, which is crucial in guiding policies and interventions to sustain their psychological well-being through a psychological crisis intervention protocol. The department of Psychological Medicine, UM, and the Counselling and Psychological Management Unit, UMMC, were mandated to develop a psychological crisis intervention protocol in the context of the COVID-19 pandemic, which required adherence to the hospital’s policy and guidelines.

### 1.2. Remote Psychological First Aid for Frontliners

The team decided a Psychological First Aid (PFA) protocol as the appropriate intervention, based on assisting people in the immediate aftermath of a disaster (natural, man-made, or infectious disease outbreaks), mitigating overwhelming acute emotional reactions, promoting safety, calmness, and hope, and assessing basic psychological and social needs to foster short- and long-term adaptive functioning [10,11,12]. The PFA was originally designed as an evidence-informed approach with lesser risk of adverse psychological impact as compared to debriefing [10], which was described by WHO [10] as “a humane, supportive response to a fellow human being who is suffering and may need support”.

Considering the contagious and persisting nature of COVID-19, the team must comply to strict physical distancing, hence replacing face-to-face interventions with tele-psychiatry via the WhatsApp mobile application and phone calls (termed remote PFA in this article). Guidelines adapted from the “Remote Psychological First Aid during the COVID-19 outbreak” by the International Federation of Red Crescent Societies [13] were used. This guideline apply the principles of “Look, Listen, Link” using phones and an on-line platform, which include (1) assessment of the current risk and status of the HCW following referral; (2) empathic listening during phone calls to allow the HCW to overcome emotional difficulties as well as to collect relevant information about his/her needs and concerns; (3) providing a link to a social support system and access to further intensive care as needed. 

### 1.3. Stepwise Remote PFA Framework for SMART Output, Outcome, and Aim

Because of the time constraint, the authors decided to adopt a PFA framework based on stepwise implementation within a healthcare system to achieve the outlined objectives [11]. Although, originally, this theory-based concept focuses on the pre-event planning and evaluation processes, we decided to apply this stepwise PFA framework carrying out the evaluation or review of the protocol at the final stage. Considering the protracted nature of the outbreak, this protocol will go through re-evaluation of the whole process to ensure that its objectives continue to be met and updated and to ensure future training of new staff in the use of protocol. Forbes et al. [11] stated that the principles of PFA can be applied within an organizational setting defined as high-risk “in which exposure to psychological trauma is either a possible or, indeed, a predictable event”, and UMMC fits into these criteria even before the COVID-19 pandemic. As the organization systematically and synergistically coordinates measures to contain the spread of infection, this has undoubtedly triggered a widespread fear and panic among the frontliners in protecting themselves and their families from being infected. 

**Figure 1:**
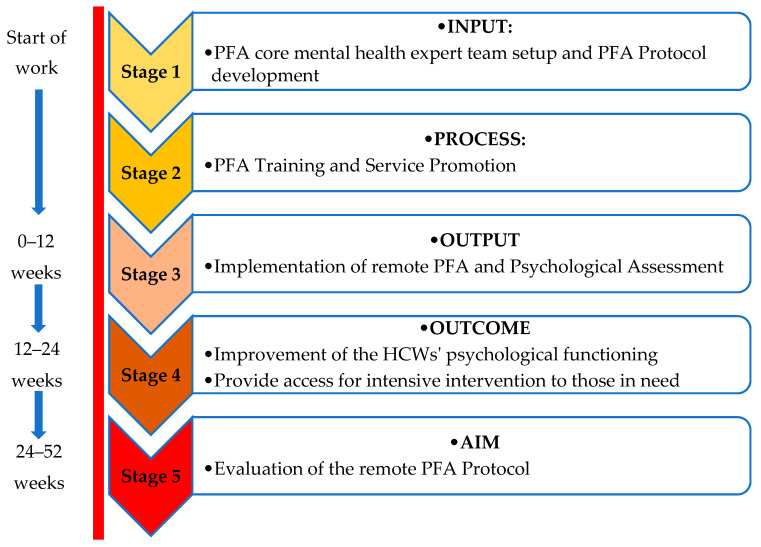
Hierarchy of goals of the remote Psychological First Aid (PFA) protocol for Healthcare Workers (HCWs) to be achieved in stages within a set timeframe during the COVID-19 pandemic in University Malaya Medical Centre (UMMC) (adapted from Ogbeiwi, 2017).

Another approach that was also adopted in the development of the protocol is the “goal-setting approach” that is defined as “the desired end result of an action that is expected to be achieved at some specified time in the future and toward which all effort and essential resources are committed” [14]. The process leading to the achievement of the objectives is based on the conceptual SMART framework (Specific, Measurable, Attainable, Relevant, Realistic Timeframe), which serves as a tool to determine if an intervention is on its planned course [15] as well as to evaluate the overall success of an intervention [15,16]. This approach has been applied in clinical work as a collaborative effort with pediatrics and aphasia rehabilitation patients [17] and patients with psychiatric disability [18], and has shown to promote the attainment of clinical goals [19]. In order to develop an evidence-informed PFA protocol, we adapted a goal setting formulated by Ogbeiwi [20] in a health context, evaluating goal accomplishment at three levels: stage 3 or output (immediate goal), stage 4 or outcome (intermediate goal), and stage 5 or impact expressed as aim (long-term goal) (Figure 1). 

This article describes the process of developing a remote Psychological First Aid protocol with appropriate adaptation that fulfils the following objectives:

i. to provide PFA training to mental health experts (psychiatrist, psychologist, counsellor) as PFA providers based on COVID-19 PFA guidelines;
ii. to provide early remote PFA to consenting frontliners via telepsychiatry;
iii. to encourage healthcare workers to get psychological help through online promotion and awareness campaigns and to minimize stigma;
iv. to measure quantitatively the level of depression, anxiety, distress, and burnout in frontliners through online assessment while maintaining confidentiality;
v. to provide access to more intensive intervention for those who require it;
vi. to review and evaluate the protocol within a stipulated time frame.

## 2. Materials and Methods 

Literature searches were carried out on the psychological crisis experienced by HCWs during the COVID-19 pandemic, with emphasis on the current state of the outbreak and practical considerations by the core team members. The papers were drawn from existing evidence-based guidelines in PubMed and Google Scholar, using the keywords: “COVID-19”, “coronavirus”, “acute psychological interventions”, “healthcare workers”, “Psychological First Aid”, “goal-directed framework”, and “SMART”. Evidence was extracted from the literature, with verification of the relevant existing and latest organizational policies, data management, and confidentiality, the use of an official online system, the clinical and nonclinical staff categories necessary for the intervention, and the proposed timeframe to achieve the objectives. The criteria for the HCWs to receive a psychological intervention were: being a frontliner worker in the COVID-19 pandemic and being a positive case or under investigation or surveillance for suspected infection due to close contact with COVID-19-positive cases. These evidence-informed data directed our focus on (1) PFA guideline development based on the existing Malaysian Ministry of Health guidelines and WHO standard protocol, as well from other recent credible sources that are accessible online and COVID-19-related; (2) identification of mental health experts (psychiatrists, psychologists, counsellors) for each stage of the work process and goal-directed activities; (3) valid mental health assessment tools to be used via the online platform, reliable before and after receiving an intervention; (4) safeguarding the confidentiality through a data management protocol; (5) modes of remote PFA training that covered fundamental aspects of PFA while complying to physical distancing; and (6) service promotion to encourage participation and minimize stigma. 

### 2.1. Protocol Development for Remote PFA: Stepwise Process Implementation and Expected Immediate, Intermediate, and Long-Term Goals

The protocol clearly articulates the work process and activities in stage 1 and 2 and establishes expected goals that conform to the five essential principles of psychological crisis interventions, promoting (1) a sense of safety, (2) calm, (3) a sense of self- and collective efficacy, (4) connectedness, and (5) hope [21].

#### 2.1.1. Stage 1: Input—PFA Core Mental Health Expert Team Set-Up and Remote PFA Protocol Development

At this stage, the team led by the head of the Psychological Medicine department, selects the core mental health expert team to be assigned to research and development, PFA training, data management, and mental health promotion related to COVID-19. The needs and practical aspects of the proposal are identified to establish the objectives:

1. Identification of the needs of the HCWs: All identified HCWs who consented to the remote PFA are assessed to make sure that they have access to the WhatsApp mobile application for referral to be made to the PFA Provider and for on-line psychological screening (pre- and post-PFA). Any issues regarding accessibility to the service will be managed by the Task Force sub-committee.
2. Identification of the needs of the PFA providers: The hospital provides a mobile phone for the PFA provider on duty. WhatsApp messages will be used for initial communications by the provider to introduce the protocol and develop an interaction with the HCW. Once the rapport is established, the mode of communication will be through phone calls that can be either a one-off call or follow-up calls, depending on the HCW’s needs.
3. Sufficient number of mental health experts as PFA providers: A total of 14 psychiatrists, 6 psychologists, and a counsellor agreed to participate, allowing a daily schedule of interventions for 12 weeks.
4. Identification of PFA trainers and sessions: three PFA trained mental health experts were identified who were responsible for half-a-day training.
5. Realistic time frame: The hospital management orders the rescheduling of psychiatrist, psychologist, and counsellor ambulatory services for 12 weeks, which allows the PFA providers to focus on the PFA services within the stipulated time frame.
6. Identification of the measurement tool: The team agreed on the use of the DASS-21 (Depression, Anxiety, Stress Scale) and CBI (Copenhagen Burnout Inventory) as the psychological assessment tools pre- and post-PFA.

Confidentiality of the HCWs requiring remote PFA is safeguarded by a data manager from the team who manages the Central Data Collation Unit (CDCU) which receives data from the Occupational Safety and Healthy Environmental unit (OSHE), Public Health department, and Staff Health unit, UMMC. 

#### 2.1.2. Stage 2: Process—Intensive Remote PFA Training and Service Promotion

In stage 2, the process involves concurrent training for remote PFA providers and promotion of the service via the hospital’s website, social media, and posters. Half-a-day training for mental health experts includes explanation of the PFA concept, utilizing WhatsApp mobile application and phone calls, and modified role-playing based on the remote PFA guideline. The most important aspect of the training involves the following:

1. The use of WhatsApp mainly for seeking immediate clarification and permission to initiate the session immediately after referral from the CDCU;
2. The use of phone calls as a means of conducting and terminating a session;
3. The need of the PFA provider to develop the skill of empathic and reflective listening during conversations via phone calls, especially when dealing with acutely distressed, angry, or confused HCWs.
4. Maintaining personal and verbal conduct throughout the sessions since both the provider and the HCW may have to overcome own difficulties, such as possible tensions which require the provider to stay calm, despite her/his own fear of COVID-19.
5. Understanding the aim of PFA, which is to promote safety, calm, hope, and connectedness, in contrast to debriefing [13].
6. Introduction of the eight-core actions of PFA as part of the “Look, Listen, Link” principle, adapted from the conventional face-to-face PFA [11]). These elements of the PFA are introduced as an overview of the PFA core actions to enhance knowledge and highlight the practical aspects of these PFA principles prior to implementation during the COVID-19 outbreak (Table 1).

The PFA providers become subsequently part of the Psychosocial Support Team (PST), and each member receives a letter of appointment from the hospital director. Relevant information on the psychosocial impact of COVID-19 and symptoms of psychological distress, as well as a link for psychosocial assistance are provided via the official website and portal to create awareness among the staff that the organization is committed to mental health care during COVID-19 outbreak and indirectly minimizes stigma related to seeking care for mental health issues.

#### 2.1.3. Stage 3: Output—Implementation of Remote PFA, Psychological Assessment of the Frontliners

The PFA providers must act in ways that respect the safety, dignity, and rights of the fellow HCWs. All frontliners that fulfil the criteria for a PFA intervention are identified and give informed consent regarding three disciplines under UMMC that have been tasked to their respective experts (OSHE unit, Public Health department, Staff Health unit), and their personal data (name, position, affiliation, COVID status, contact number) are channeled by the data manager to the PFA providers, according to the duty roster. The PFA providers must contact the HCW within 24 h to deliver the task according to the guidelines and training received in Stage 2. Qualitative interviews exploring elements of the remote PFA experience, preparedness, and suggestions for improving the delivery of the service are conducted among the PFA providers following verbal consent.

Each HCW is invited to complete the mental health screening that is available online via a Google form, which utilizes the DASS-21 and the CBI scoring. DASS-21 is a set of three self-reported questionnaires designed to measure the negative emotional states of depression, anxiety, and stress in individuals [22]; the CBI consists of three scales that measures personal burnout, work-related burnout, and client-related burnout [23]. Both DASS-21 and CBI are validated and have shown high sensitivity and specificity in their psychometric properties for use in health-care professional settings. The advantage of these scales is that they are self-reported scales and are feasible to be administered remotely. The CDCU manager is tasked with collating all mental health data under strict confidentiality [24].

In stage 3, the immediate goal is established, with the following output:

1. Specific output: The PFA providers can provide telepsychiatry interventions confidently and responsibly within 24 h of receiving referral via WhatsApp. The HCWs that fulfil the criteria for referral to PFA providers consented to the intervention and undergo a mental health screening using DASS-21 and CBI.
2. Measurable output: The level of depression, anxiety, distress, and burnout of the HCWs are measured immediately upon referral via the online form and 2 weeks after PFA completion.
3. Attainable output: Each HCW is distributed by the CDCU manager in a 1:1:1 ratio among the psychiatrists, psychologists, and counselors.
4. Relevant and realistic timeframe output: The remote PFA intervention is still relevant due to the protracted nature of the COVID-19 pandemic. The establishment of a short-term goal within 12 weeks is practical and realistic.

#### 2.1.4. Stage 4: Outcome—Improvement of the HCWs’ Psychological Functioning and Accessibility to Intensive Interventions for Severe Cases

Stage 4 is the establishment of the overall objective goals and is considered an intermediate stage of goal achievement. The outcome may not be observed immediately but may take place when there are changes or improvement in the organization policy and commitment towards mental health care, increase in help-seeking behavior among HCWs, increased awareness of the importance of attaining psychological and occupational functioning despite having previously been treated for COVID-19, hence reduction of stigmatization towards these HCWs by fellow colleagues. For those showing a significant impact on their mental health based on DASS-21 or/and CBI, a further assessment and treatment by a psychiatrist or psychologist may be necessary, or in case of practical needs, by social welfare personnel. A timely intervention must be made available for the patient to return to baseline occupational and inter-personal functioning.

The expected intermediate goal of stage 4 is expected to have the following outcome:

1. Specific outcome: All HCWs who consented to the remote PFA intervention are attended to by mental health experts (PFA providers) via WhatsApp and phone calls. All HCWs with severe psychological and social needs are referred to relevant experts.
2. Measurable outcome: The data collected from the CDCU on HCWs’ DASS-21 and CBI are analyzed quantitatively for the psychological impact of COVID-19 before and after the intervention. The level of psychological distress and/or burnout will improve between 30% to 50%. (At the time of writing, the process is still ongoing).
3. Attainable outcome: The number of mental health experts (psychiatrists, psychologists, and counsellors) to provide psychosocial crisis interventions for HCWs is adequate and is supported by the organization top management.
4. Relevant and realistic timeframe outcome: A period of 12 weeks is considered realistic to achieve all the objectives successfully, and due to the protracted nature of COVID-19, the remote PFA may remain relevant up to 24 weeks.

#### 2.1.5. Stage 5: Aim—Evaluation of the Remote PFA Protocol

At the time of writing, the team has managed to establish the stage 3 output and is in the process of analysis of pre- and post-PFA data [22]. According to Forbes et al. [11], a feasibility assessment is recommended, although it demands much effort and undertaking from the organization via a pre-, post-, and follow-up quantitative analysis of individual psychological distress. Ogbeiwi (2018) suggested a period between 12 weeks to 1 year to achieve a short-term outcome related to the objectives and of at least 5 years to determine the long-term impact of the aim. As for the remote PFA in the context of COVID-19, one year would be practical to establish the impact of COVID-19; this period may be revised according to the latest development of the pandemic, based on directives from the Task Force.

In stage 5, the expected long-term goal will have the following aims:

1. Specific aim: Every stages of the protocol is evaluated by experts from Psychological Medicine, Occupational Safety and Environmental Health, Public Health, Nursing, as well as administrative representatives from UMMC with regard to the work process, data management, and preparedness of the mental health experts (PFA providers and trainers).
2. Measurable aim: Validated tools are identified by experts in epidemiology and industrial and organization psychologists to specifically assess the work-related distress caused by the COVID-19 pandemic.
3. Attainable aim: Virtual psychological interventions within the boundaries of medical ethics and hospital policy are developed by information technology experts, mental health experts, legal advisors, and representatives of stakeholders identified by UMMC.
4. Relevant and realistic timeframe aim: An action plan that includes a feasibility study of remote psychological crisis interventions using a virtual technology protocol may further improve the training and service delivery within the next two to four years.

## 3. Discussion

The protocol for psychological intervention that employs a mobile application and phone call known as remote PFA was developed for the first time at the University Malaya Medical Centre, which is the first non-MoH hospital to be designated as COVID-19 hospital. Although PFA guidelines are available both locally under MoH [25] and internationally [10,11] for use in the immediate aftermath of traumatic events or disasters, PFA during the COVID-19 pandemic is scarce and was developed for use with public, vulnerable groups such as children and elderly and has limited efficacy for healthcare workers or frontliners. 

The protocol here developed was built with the general objective of providing psychological and social support to frontliners affected by the disease or suspecting to be infected and waiting for the swab test result, who remain in self-quarantine for a duration of 14 days, to promote their psychological recovery. Within days after starting developing the protocol, we were able to complete a literature research and unanimously agreed to adapt the protocol by the International Federation of Red Cross and Red Crescent Societies (2020), entitled “Remote Psychological First Aid during the COVID-19 outbreak” [13]. This guidelines underwent minor changes conforming to cultural and practical features of Malays healthcare workers, while preserving the core elements of PFA “Look, Listen, Link” which promote a sense of safety, self- and collective efficacy, calm, connectedness, and hope [11,21,26].

Formulating appropriate goals for healthcare organizations is undoubtedly essential to build programs, especially for result-oriented organization or “high-risk organization” that deal with acute emergencies either physically or psychologically [11]. The SMART goal-directed approach was selected to enable an objective evaluation of the quality of the protocol and the progress of its implementation within an appropriate time. The SMART framework was chosen to ensure the service is delivered clearly and consistently during the COVID-19 pandemic. All interventions, progress, and updates on the overall health status of the HCWs have been compiled and measured within the stipulated timeframe of 12 weeks, prior to evaluation and final assessment of the protocol. 

We also classified the goals according to the stages of output, outcome, and aim. The first two stages are mainly devoted to forming the experts’ team and initiating the process of establishing the immediate output. The team managed to complete the task and established the output within 12 weeks of PFA inception. This unprecedented protocol for remote PFA is considered a success, with all mental health experts to working as a team and efficiently collaborating with experts of other disciplines and making a synergistic and systematic effort. It is important to recognize from the outset that mental health issues may manifest as a psychological sequela, even months to years later. As such, the protocol is designed for stage 4 (expected outcomes) and 5 (expected aim) and remains relevant after 12 weeks up to 1 year, as the country is still adapting to a “new normal” situation, both at work and in the community. PFA services, remotely for now, must be readily available to provide psychological support and accessible resources to ease the transition to normalcy based on the principle actions of contact, comfort, calm, concern, care, connection, coping, and collaboration [11].

## 4. Conclusions

This unprecedented protocol for the implementation of remote PFA allows the establishment of immediate objectives promoting psychological recovery and functioning of affected healthcare workers through early specific psychosocial interventions, utilizing valid assessment tools to measure patients’ psychological responses to the interventions, through the collaborative work of mental health experts and experts from other disciplines; it will remain relevant throughout the pandemic and beyond. This protocol that utilizes remote PFA is the first of its kind that adopts a goal-directed SMART framework in a “high-risk” university teaching hospital in Malaysia. Research that examines this distinctive approach is scarce and is much needed. Therefore, it is hoped that more research and clinical trials are carried out in the future using this “goal-directed” model. Well-formulated goals serve as a benchmark for evaluating the “relevance and overall value of policies, services, and projects at the end of implementation, which allows empirical judgement of the effectiveness, efficiency, and success of work and demonstration of management accountability for expended resources at all levels” [27].

## Figures and Tables

**Table 1:** An overview of the eight core actions based on the principle of “Look, Listen, Link” for healthcare workers during the COVID-19 pandemic (adapted from Forbes et al., 2011).

Core Actions	Targeted Goals with Remote PFA
1. Contact and Engagement	All HCWs who consented to the remote PFA intervention, are attended to by PFA providers via a WhatsApp phone call within 24 h of referral. PFA is offered once therapeutic alliance is formed, but if help is declined, respect the wish but explain that help is always available.
2. Comfort and Safety	HCWs receive immediate emotional support that promotes sense of safety, comfort, and hope.
3. Calm and Stabilization	HCWs is able to self-regulate her/his own emotions through breathing, relaxation techniques, and self-affirmative words during crises.
4. Collect Information of Current Needs and Concerns	The immediate needs and concerns of the HCWs are identified, and remote PFA is applied accordingly.
5. Care: Provide Practical Assistance	HCWs with severe psychological and social needs are referred to relevant experts.
6. Connection with Social Supports	HCWs receive continuous support from colleagues and employers without being stigmatized or discriminated.
7. Information on Coping	HCWs are able to develop positive coping skills which allow adaptive functioning and return to baseline occupational functioning.
8. Linkage with Collaborative Services	HCWs are empowered to seek further assistance from other relevant agencies such as non-governmental organizations in the community for the continuity of care.

PFA = Psychological First Aid; HCW = Healthcare workers.
